# Unipedicular vs. Bipedicular Balloon Kyphoplasty in the Treatment of Osteoporotic Vertebral Compression Fractures: Single-Institute 3-Year Follow-Up Results

**DOI:** 10.3390/medicina61040663

**Published:** 2025-04-03

**Authors:** Tolga Ege, Uğur Yüzügüldü, Ali Murat Başak, Mustafa Aydın, Ömer Erşen, Tuluhan Yunus Emre

**Affiliations:** 1 Department of Orthopedics and Traumatology, Gülhane Training and Research Hospital, 06010 Ankara, Turkey; muratalibasak@gmail.com (A.M.B.); mustafaaydin5528@gmail.com (M.A.); merschenn@yahoo.com (Ö.E.); 2 Department of Orthopedics and Traumatology, Atatürk Training and Research Hospital, 10100 Balikesir, Turkey; uguryuzuguldu@gmail.com; 3 Department of Orthopedics and Traumatology, Dr. Şinasi Can Kadıköy Acıbadem Hospital, 34718 Istanbul, Turkey; tuluhan.emre@acibadem.com

**Keywords:** osteoporosis, balloon kyphoplasty, vertebrae compression fractures

## Abstract

*Background and Objectives:* Balloon kyphoplasty is one of the most commonly performed minimally invasive surgical procedures for the treatment of osteoporotic vertebral fractures, with the bipedicular technique being the conventional approach. However, the use of both pedicles may present certain disadvantages, including higher costs, longer operative times, increased radiation exposure, and a greater risk of bone cement leakage. This study aims to report the 3-year follow-up outcomes of double-pedicle and single-pedicle kyphoplasty performed at our institution. *Materials and Methods:* Between June 2016 and May 2019, a total of 136 patients who presented to our clinic with osteoporotic vertebral fractures and underwent balloon kyphoplasty were included in this retrospective study. Pain relief and quality of life indices were assessed preoperatively and postoperatively. During follow-up examinations, radiographs, VAS (Visual Analog Scale) scores, and ODI (Oswestry Disability Index) scores were evaluated. Radiation exposure was assessed using fluoroscopy time and dose area product (DAP) values. Additionally, total injected cement volume, operative time, and procedural complications were retrieved from patient records. *Results:* The procedure was successful in all patients. The mean bone cement volume used was 3.4 ± 1.4 mL in the unipedicular group and 5.3 ± 2.1 mL in the bipedicular group. Fluoroscopy time and DAP values were significantly higher in the bipedicular technique compared to the unipedicular technique. At the final follow-up, the average kyphosis correction and mean vertebral height correction ratio were greater in the bipedicular group. The mean reduction in VAS and ODI scores was superior in the bipedicular group at the 1-, 2-, and 6-month follow-ups. However, at the 1-, 2-, and 3-year follow-ups, there was no significant difference in VAS and ODI scores between the two groups. *Conclusions:* The unipedicular balloon kyphoplasty technique offers several advantages, including shorter operative time, lower cement leakage risk, reduced radiation exposure, and comparable pain score reductions at 1- to 3-year follow-ups. However, the bipedicular technique provides superior short-term pain relief and demonstrates better sagittal alignment correction in long-term follow-ups compared to the unipedicular approach.

## 1. Introduction

Osteoporotic vertebral compression fractures (OVCFs) are most commonly observed in the elderly population and can significantly increase morbidity and mortality [1]. These fractures cause severe pain, leading to a decline in quality of life and a reduction in vertebral height, ultimately resulting in spinal imbalance [2]. Moreover, prolonged bed rest due to these fractures may further decrease bone density, predisposing patients to new fractures and pressure ulcers. As spinal deformity progresses, nutritional disorders, depression, and impaired pulmonary function may also develop [1].

Conventional conservative treatment options for these fractures include bed rest, bracing, physical support, and analgesic therapy. However, despite these treatments, progressive vertebral collapse and persistent pain may still occur in some cases [1,2]. Historically, vertebroplasty (VP) was initially introduced for treating vertebral hemangiomas and later adapted for spinal fractures. However, vertebroplasty has certain limitations, including inadequate vertebral height restoration and the risk of bone cement leakage [3].

Percutaneous kyphoplasty (PKP) involves accessing the fractured vertebral body through the pedicle, inflating a balloon within the vertebral body to create a cavity, and subsequently injecting semi-solid bone cement to stabilize the fracture [4]. Polymethylmethacrylate (PMMA) bone cement is used in this procedure, providing immediate pain relief, improved mobility, significant reduction in morbidity, and restoration of both vertebral height and kyphotic angle (KA) in patients with osteoporotic vertebral compression fractures.

Although the traditional approach in balloon kyphoplasty involves the bipedicular technique, studies have reported that similar clinical and radiological outcomes can be achieved using the unipedicular approach, with the added benefits of reduced operative time, lower cement volume, decreased costs, and reduced radiation exposure [5,6]. Despite recent meta-analyses, no definitive consensus has been established regarding the superiority of either the unipedicular or bipedicular approach. Osteoporotic vertebral fractures remain a significant clinical concern, and the debate regarding the advantages of these techniques continues in the literature. This study aims to compare the three-year follow-up outcomes of balloon kyphoplasty performed using unipedicular versus bipedicular techniques at our institution.

## 2. Patients and Methods

### 2.1. Study Design

The Institutional Review Board of the local ethics committee approved this retrospective study (date: 19 January 2024, number: 2024-160). Between June 2016 and May 2019, a total of 136 patients with osteoporotic vertebral compression fractures (OVCFs) who underwent percutaneous kyphoplasty using either a unipedicular or bipedicular approach were included in this retrospective study. The patients were categorized into two groups: the single-pedicle extra-pedicular approach group and the bipedicular transpedicular approach group.

Patient selection criteria: Patients over 50 years of age who did not respond to conservative treatment during the 6-week subacute fracture period, had a VAS score of 6 and above (to include patients with clinically significant pain who were most likely to benefit from surgical intervention), and showed edema at the fracture site on MRI t2, and fat-suppressed sequences were selected. Patients with pathological fractures, multiple-level osteoporotic fractures (to ensure a more homogeneous study population and minimize confounding factors), VAS (Visual Analog Scale) scores lower than 6 with conservative treatment, patients with bleeding disorders, local infections, and patients who did not have sufficient follow-up time were excluded from the study.

### 2.2. Surgical Procedure

Bipedicular group: The patients were placed in the prone position on the radiolucent surgical table. Soft surgical pads were placed on the sternum and pelvis to help correct kyphosis. After aseptic cleaning and covering the surgical field with a surgical drape, lidocaine was injected into the pedicle area and soft tissues under fluoroscopy control with a number 22 needle. Then, the trocar was medialized 10–20 degrees in the anterior–posterior plane and sent through the pedicle towards the vertebral body. The same procedure was repeated for the opposite pedicle. Then, the working cannula was advanced over the guidewire to the posterior 1/3 part of the vertebral body. Then, the balloon catheter was sent into the vertebral body through the working cannula, inflated bilaterally, and a sufficient amount of semi-solid bone cement was injected into both sides.

**Figure 1:**
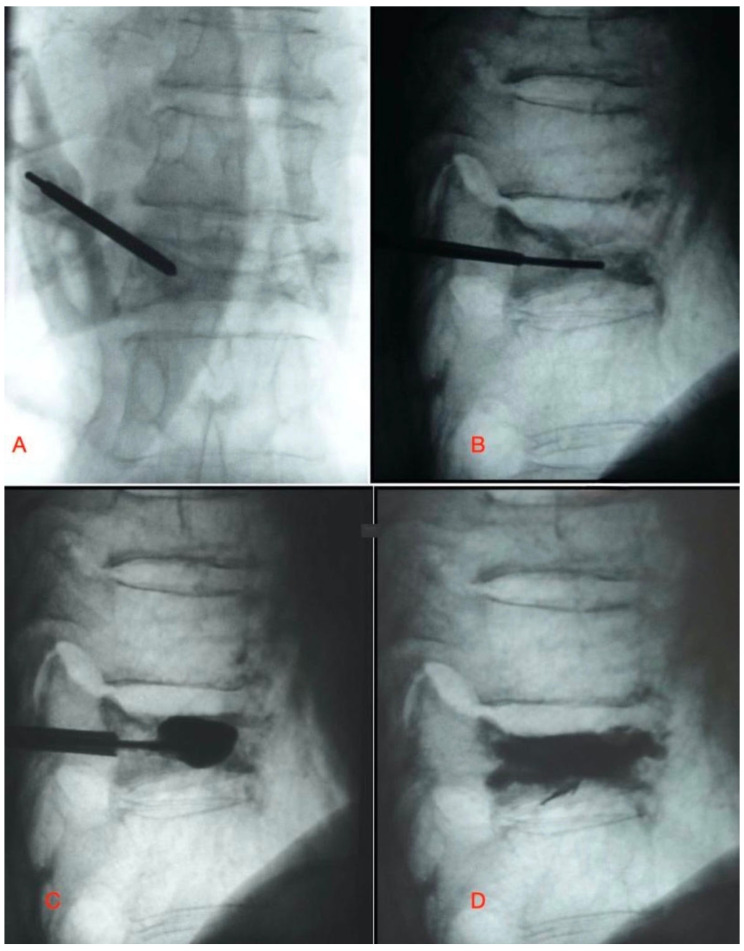
(**A**) The trocar was inserted in the extrapedicular site and medialized 20–30 degrees in the anterior–posterior plane, and it was advanced to posterior 1/3 of the vertebral body. (**B**) The working cannula was inserted through the guidewire. (**C**) It was advanced to the anterior 2/3 of the vertebral body, aligning it with the central part of the body as much as possible. (**D**) The balloon was inflated, and semi-solid bone cement was injected.

Unipedicular group: Patients were positioned similarly to the bipedicular group. After aseptic cleaning and covering the surgical field with a surgical drape, lidocaine was injected into the pedicle area and soft tissues under fluoroscopy control with a number 22 needle. Subsequently, the trocar was centrally placed in the vertebral body at an angle of 20° to 30° to the AP axis, using an extrapedicular approach under fluoroscopy. After inserting the guidewire, the cannula was introduced laterally to the posterior third of the vertebral body. The same balloon catheter was used in the unipedicular technique; the trocar was medialized 20–30 degrees in the anterior–posterior plane, and it was advanced to the anterior 2/3 of the vertebral body, aligning it with the central part of the body as much as possible (Figure 1). After the confirmation of the cannula by fluoroscopy, the balloon was inflated, and semi-solid bone cement was injected.

All surgeries were performed by 2 surgeons with at least 5 years of experience in spinal surgery. Patients were closely monitored at the hospital for at least 24 h post-surgery. Vital signs and any potential complications were assessed.

### 2.3. Data Collection

The demographic data included age, gender, comorbidities, bone mineral density (BMD), time from pain onset to surgery, history of cancer, diabetes, pulmonary and renal disorders, and thyroid dysfunction.

### 2.4. Clinical Evaluation

VAS and ODI scores were assessed by independent physicians who were not directly involved in the surgeries. However, the assessors were not explicitly blinded to the surgical technique, which may introduce a potential source of measurement bias. Pain scores were rated on a scale from 0 to 10, with 0 indicating ‘no pain’ and 10 representing the ‘most severe pain.’ VAS and ODI (Oswestry Disability Index) scores were recorded preoperatively, immediately postoperatively, and at 1, 3, 6, and 12 months, as well as at 2 and 3 years postoperatively.

### 2.5. Radiological Evaluation

**Figure 2:**
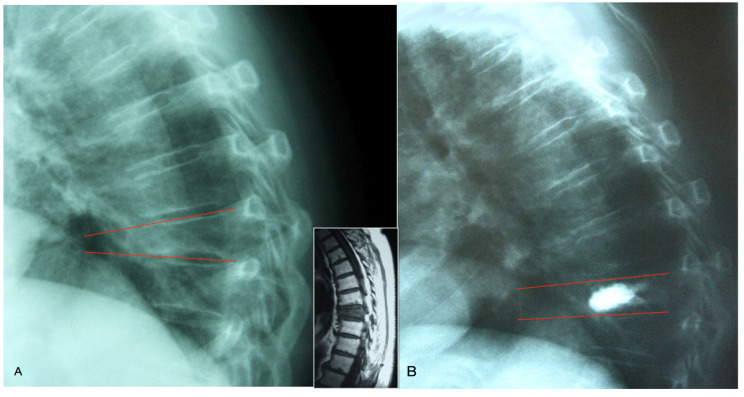
Measuring the kyphotic angle. It was calculated as measuring the angles between lines running parallel to the upper and lower endplates of fractured vertebrae before and after surgery (**A**,**B**) (Sagittal MRI showing bone edema due to acute fracture).

**Figure 3:**
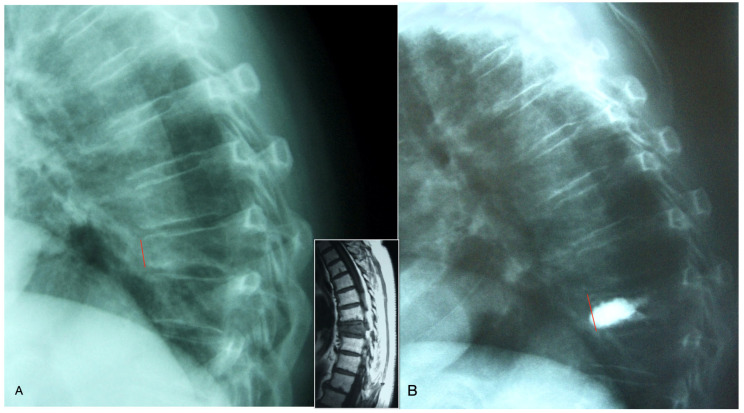
Recovery of the vertebral height was measured by measuring the lowest vertebral height before and after surgery. (**A**) The arrow indicates the lowest vertebral height. (**B**) The arrow indicates the recovery of the vertebral height at the same location after surgery (Sagittal MRI showing bone edema due to acute fracture).

For radiological evaluation, all patient radiographs were retrieved from the hospital’s PACS (Picture Archiving and Communication System), and distance and angle measurements were performed digitally. Printed radiographs were not used due to the potential for magnification errors. The kyphosis angle (KA) (Figure 2) and the lowest anterior vertebral height of the fractured vertebra (Figure 3) were measured preoperatively, immediately postoperatively, at two years, and at three years, with the difference in healing rates calculated. The kyphosis angle (KA) was determined by measuring the intersection angle between lines parallel to the upper and lower end plates of the fractured vertebra. The lowest anterior vertebral body height was measured from the shortest region of the anterior vertebral wall. Postoperative vertebral height measurements were obtained from the same location on follow-up radiographs. Changes in kyphosis angle and anterior vertebral height were measured and are expressed as a proportion relative to the initial values.

### 2.6. Surgical Evaluation

In the surgical evaluation, the duration of surgery, complications, and the amount of bone cement injected were discussed. Postoperative complications were any bone cement leakage outside the vertebral body, infection, and adjacent vertebral compression fracture (AVCF) that could be detected during follow-up.

### 2.7. Statistical Analysis

The IBM SPSS for Windows 20.0 (SPSS Inc., Chicago, IL, USA) program was used in the evaluations, and *p* < 0.05 was accepted as the limit of statistical significance. In statistical analysis, a Student’s *t*-test (significance test of the difference between two means) was used for parametric data, and Mann–Whitney U test was used for non-parametric data. A repeated measures ANOVA was used to assess changes over time within groups. Post hoc pairwise comparisons were adjusted for multiple testing. An a priori power analysis was not conducted, which may impact the statistical power of our findings.

## 3. Results

We reviewed the medical records of 158 patients who were admitted to our center with vertebral compression fractures. Patients with pathological fractures (e.g., vertebral metastases) and those who underwent multiple kyphoplasty (KP) or vertebroplasty (VP) due to multilevel fractures were excluded from the study.

After applying the exclusion criteria, 136 patients with osteoporotic vertebral compression fractures were included in the final analysis. There were no statistically significant differences between the two groups in terms of baseline characteristics (Table 1).

### 3.1. Clinical Findings

VAS and ODI scores at 1, 3, 6, and 12 months, as well as at two and three years, were significantly lower in both groups compared to preoperative values.

At the 12-month, 2-year, and 3-year follow-ups, there were no significant differences in VAS and ODI scores between the two groups when accounting for patient variables. However, the bipedicular group had significantly lower VAS and ODI scores at the 1-, 2-, and 6-month follow-ups compared to the unipedicular group.

Beyond 12 months, and in subsequent follow-up periods, there was no significant difference in clinical scores between the two groups (Table 2 and Table 3).

### 3.2. Radiological Analysis

Intra- and inter-observer reliability for radiological measurements (kyphosis angle and vertebral height) was assessed using intra-class correlation coefficients (ICCs). The intra-observer ICC was 0.92, and the inter-observer ICC was 0.89, indicating excellent reliability.

All radiological parameters, including kyphosis angle and lowest vertebral height, improved following surgery. However, in the unipedicular group, vertebral height loss and kyphotic deformity were observed at the 2- and 3-year follow-ups, particularly in the lumbar region, where the deterioration was more pronounced than in the thoracic region (Table 4 and Table 5).

Lateral wedging was detected in three patients in the unipedicular group, all of whom had fractures in the lumbar region. Notably, radiological worsening in the unipedicular group was not associated with a decline in clinical scores.

### 3.3. Surgical Results

Regarding surgical duration, the unipedicular technique resulted in significantly shorter operative times compared to the bipedicular technique (22.7 ± 4.6 min vs. 32.6 ± 9.6 min; *p* = 0.003).

Similarly, the volume of polymethylmethacrylate (PMMA) injected was significantly lower in the unipedicular group than in the bipedicular group (3.4 ± 1.4 mL vs. 5.3 ± 2.1 mL; *p* = 0.002). This difference in PMMA volume is directly related to the surgical approach. In the unipedicular group, a single entry point restricts the distribution and total amount of cement injected. In contrast, the bipedicular technique allows for more symmetrical cement distribution, leading to a higher total volume. This finding is consistent with those of previous studies that emphasize the role of cement volume in pain relief and vertebral height restoration.

Fluoroscopy time and effective radiation dose were also significantly lower in the unipedicular group compared to the bipedicular group (1.7 ± 3.1 min vs. 2.6 ± 1.1 min; *p* = 0.003). The dose area product (DAP) values were 330.98 ± 38.3 cGy·cm^2^ in the unipedicular group and 515.66 ± 43.1 cGy·cm^2^ in the bipedicular group (*p* = 0.002)

### 3.4. Complications

No cases of bone cement embolism to the lungs, major vessels, or epidural space were observed in either group.

Cement leakage into the intradiscal space occurred in three patients in the unipedicular group and five patients in the bipedicular group, with no significant difference between the groups (*p* = 0.67). None of these patients exhibited neurological deficits or clinical symptoms related to cement leakage.

Additionally, there was no significant difference in the occurrence rate of adjacent vertebral compression fractures (AVCFs) between the two groups postoperatively (*p* = 0.72). AVCFs were observed in 12 of 70 patients in the unipedicular group and 19 of 66 patients in the bipedicular group.

## 4. Discussion

This study aimed to compare the outcomes and postoperative complications between patients who underwent unipedicular and bipedicular balloon kyphoplasty for osteoporotic vertebral compression fractures (OVCFs).

Osteoporosis leads to the progressive deterioration of vertebral body integrity and is characterized by a bone mineral density (BMD) 2.5 or more standard deviations (SD) below the mean of a healthy young population. As a result of this metabolic disorder, vertebral compression fractures may occur due to altered biomechanical forces acting on the vertebral cortex [7].

The first clinical results of percutaneous kyphoplasty (PKP), reported by Lieberman et al. in 2001, demonstrated several advantages over vertebroplasty (VP). Specifically, PKP provides better vertebral height restoration and has a lower incidence of bone cement-related complications compared to vertebroplasty [8].

Although the traditional approach for percutaneous kyphoplasty (PKP) is the bipedicular technique, the unipedicular technique is also widely utilized due to several advantages, including shorter operative time, reduced radiation exposure, and lower puncture site-related complication risks [1].

A key advantage of the unipedicular technique over the bipedicular approach, as reported in the literature, is that clinical outcomes remain comparable. Papadopoulos et al. compared the 1-year short-term clinical outcomes of unipedicular PKP with the bipedicular technique and found no significant differences in VAS, ODI, and SF-36 scores [9]. Similarly, Li et al. conducted a meta-analysis evaluating VAS scores and reported that both short- and long-term clinical outcomes were similar between the two techniques [10].

Additionally, their study found that, although more cement was used in the bipedicular technique, it did not have a significant impact on pain relief [10]. In our study, bone cement volume was also higher in the bipedicular group, and superior pain relief was observed within the first six months postoperatively

Contrary to Li et al., we believe better pain relief with the bipedicular technique in short-term follow-ups depends on the higher amount of bone cement. Similar to our study, Röder et al. stated that, in PKP, one of the most critical factors in pain relief is the amount of bone cement, and they recommended using bone cement of 4.5 mL or above per level for a successful clinical result [11]. One of the essential results we found in our study is that, although the radiological recovery was similar for up to 1 year in both groups, vertebral height restoration was better maintained with the bipedicular technique in longer follow-ups. In the meta-analysis of Li et al., no difference was found in radiologic outcomes performed with unipedicular and bipedicular techniques in seven RCT [1]. In the study of Chen et al., the restoration rate in the bipedicular group was higher than in the unipedicular group (*p* = 0.005) in the early stage, and the height lost ratio showed no significant differences 6 months or two years later [12]. In cadaveric studies, while the distribution of bone cement is more uniform in PKP performed with the bipedicular technique, biomechanical stability is closely related to the distribution of bone cement in the unipedicular technique [13]. Considering that our patients in both groups had similar demographic characteristics, we believe that using higher amounts of bone cement in the bipedicular group is closely associated with better long-term radiological improvement.

In our study, the unipedicular technique is more advantageous than the bipedicular technique in terms of the duration of surgery and the amount of radiation exposed. The unipedicular technique is more advantageous regarding the amount of radiation the surgeon will be exposed to and patient morbidity, especially in centers with high surgical cases. While VAS and ODI score reductions were statistically significant in early follow-ups, their clinical significance should be interpreted based on minimal clinically important difference (MCID) thresholds. Previous studies suggest an MCID of 1.5–2.0 points for VAS and 4–6 points for ODI in spinal interventions. Our findings indicate that the observed differences in short-term follow-ups may be clinically meaningful, though they diminish over time.

In our study, similar complication rates were found in both groups, and our results show similarities with the literature. Although bone cement leakage into the disc space was detected in both groups, no clinical adverse events were observed. Again, a similar risk regarding AVCFs was found in both groups.

This study has a retrospective design and includes a relatively small sample size, which may be considered a limitation. Additionally postoperative pain management protocols were standardized across groups, but data on concurrent pharmacological treatments (e.g., bisphosphonates) were not systematically recorded. This is a potential confounding factor that may have influenced pain and functional outcomes. Future studies should incorporate detailed medication tracking to account for such effects. However, the meticulous data collection in our clinic and the detailed follow-up records provide a reliable basis for the analysis. Despite these limitations, this study aims to contribute valuable insights into the comparison of unipedicular and bipedicular balloon kyphoplasty techniques, which remain a topic of ongoing debate in the literature.

## 5. Conclusions

In conclusion, the unipedicular balloon kyphoplasty technique has the advantages of a shorter operation time, lower cement leakage, lower radiation dose, and the same reduction in pain scores on 1- to 3-year follow-up exams. However, the bipedicular technique has better short-term pain scores and corrects sagittal alignment compared to the unipedicular technique on long-term follow-ups. From our experience, the choice between unipedicular and bipedicular approaches should be based on patient-specific factors: The unipedicular technique is preferable in cases where minimizing radiation exposure, reducing surgical time, and lowering cement leakage risks are priorities. It is particularly advantageous for patients with smaller vertebral body sizes, lower fracture severity, and single-level fractures where sufficient cement distribution can still be achieved through a single access point. The bipedicular technique should be considered for patients with severe vertebral height loss, significant kyphotic deformity, or when greater biomechanical stability is required. The increased cement volume and more symmetric distribution in the bipedicular technique provide better vertebral height restoration and kyphosis correction in the long term. Future prospective studies with larger cohorts are needed to further refine these indications.

## Figures and Tables

**Table 1:** Baseline characteristics of the patients (T: thoracal, L: lumbar).

	Unipedicular Group (n: 70)	Bipedicular Group (66)	*p*
Age	68.12 ± 8.4	70.3 ± 7.9	0.051
Sex (m/f)	22/48	16/50	0.069
Fracture to kyphoplasty time (days)	43 ± 11.2	38 ± 10.7	0.058
Site of compression fracture	T: 38 L: 32	T: 32 L: 34	0.062
BMD (t score)	−3.12 ± 0.6	−3.21 ± 0.58	0.057
Baseline VAS score	8.4 ± 1.2	8.6 ± 1.1	0.066

**Table 2:** Comparison of pain scores (VAS) after each time point.

Time After Surgery	Unipedicular Group	Bipedicular Group	*p*
Baseline	8.4 ± 1.2	8.6 ± 1.1	0.066
1 month	3.2 ± 0.9	2.8 ± 0.8	0.01
3 month	3.6 ± 1.1	2.7 ± 0.9	0.003
6 month	3.4 ± 1.0	2.9 ± 0.9	0.002
1 year	2.9 ± 0.8	2.8 ± 0.7	0.3
2 year	2.9 ± 0.9	2.6 ± 0.7	0.23
3 year	2.7 ± 0.7	2.6 ± 0.6	0.11

**Table 3:** Comparison of ODI scores after each time point.

Time After Surgery	Unipedicular Group	Bipedicular Group	*p*
Baseline	26.3 ± 3.2	25.1 ± 3.1	0.2
1 month	6.1 ± 1.5	4.6 ± 1.3	0.02
3 month	7.4 ± 1.6	3.56 ± 1.5	0.004
6 month	6.4 ± 1.4	4.1 ± 1.3	0.001
1 year	7.46 ± 1.3	6.5 ± 1.2	0.25
2 year	8.3 ± 1.2	7.42 ± 1.1	0.18
3 year	10.1 ± 1.5	9.62 ± 1.3	0.22

**Table 4:** Radiological results: recovery of the lowest vertebral height for each treatment group at different follow-up periods.

	Unipedicular Group (n: 70)	Bipedicular Group (n: 66)	*p*
Baseline vertebral height (cm)	13.6 ± 5.3	14.4 ± 3.3	0.58
Recovery of vertebral height immediate after surgery (cm, %)	1.96 ± 1.6 (14.4%)	2.4 ± 1.1 (16.66%)	0.12
Recovery of vertebral height after 1 year (cm, %)	1.95 ± 1.3 (14.3%)	2.2 ± 1.6 (15.2%)	0.1
Recovery of vertebral height after 2 years (cm,%)	1.70 ± 0.9 (12.5%)	2.2 ± 0.8 (15.2%)	0.001
Recovery of vertebral height after 3 years (cm, %)	1.66 ± 1.0 (12.2%)	2.0 ± 1.3 (13.8%)	0.002

**Table 5:** Recovery of kyphotic angles or each treatment group at different follow-up periods.

	Unipedicular Group (n: 70)	Bipedicular Group (n: 66)	*p*
Baseline kyphotic angle	13.6 ± 3.1	13.8 ± 2.7	0.8
Recovery of kyphotic angle immediate after surgery/percentage	3.6 ± 1.8(26.4%)	4.1 ± 1.4(29.7%)	0.6
Recovery of kyphotic angle after 1 year/percentage	3.4 ± 1.1/(25%)	3.9 ± 1.3/(28.2%)	0.56
Recovery of kyphotic angle after 2 years/percentage	2.8 ± 1/(20.5%)	3.6 ± 1.2/(26%)	0.03
Recovery of kyphotic angle after 3 years/percentage	2.6 ± 1.1/(19.11%)	3.3 ± 1.3 /(23.9%)	0.02

## Data Availability

The data presented in this study are available on request from the corresponding author. The data are not publicly available due to privacy restrictions.
